# HHT diagnosis by Mid-infrared spectroscopy and artificial neural network analysis

**DOI:** 10.1186/1750-1172-8-94

**Published:** 2013-06-27

**Authors:** Andreas Lux, Ralf Müller, Mark Tulk, Carla Olivieri, Roberto Zarrabeita, Theresia Salonikios, Bernhard Wirnitzer

**Affiliations:** 1 Q-bios GmbH Biotechnology, Mannheim, Germany and Faculty of Medicine at Mannheim, University of Heidelberg, Mannheim, Germany; 2 Institute of Instrumental Analysis and Bioanalysis, Department of Biotechnology, Mannheim University of Applied Sciences, Mannheim, Germany; 3 Department of Molecular Medicine, University of Pavia, Pavia, Italy; 4 Hospital Sierrallana, Centro de Referencia HHT, Cantabria, Torrelavega, Spain; 5 Institute of Digital Signal Processing, Mannheim University of Applied Sciences, Mannheim, Germany

**Keywords:** ALK1, Artificial Neural Network, Diagnostic, Disease, Endoglin, Hereditary Hemorrhagic Telangiectasia, Mid-infrared Spectroscopy, Mutation

## Abstract

**Background:**

The vascular disorder Hereditary Hemorrhagic Telangiectasia (HHT) is in general an inherited disease caused by mutations in the TGF-β/BMP receptors endoglin or ALK1 or in rare cases by mutations of the TGF-β signal transducer protein Smad4 leading to the combined syndrome of juvenile polyposis and HHT. HHT is characterized by several clinical symptoms like spontaneous and recurrent epistaxis, multiple telangiectases at sites like lips, oral cavity, fingers, nose, and visceral lesions like gastrointestinal telangiectasia, pulmonary, hepatic, cerebral or spinal arteriovenous malformations. The disease shows an inter- and intra-family variability in penetrance as well as symptoms from mild to life threatening. Penetrance is also depending on age. Diagnosis of the disease is based on the presence of some of the listed symptoms or by genetic testing. HHT diagnosis is laborious, time consuming, costly and sometimes uncertain. Not all typical symptoms may be present, especially at a younger age, and genetic testing does not always identify the disease causing mutation.

**Methods:**

Infrared (IR) spectroscopy was investigated as a potential alternative to the current diagnostic methods. IR-spectra were obtained by Fourier-transform Mid-IR spectroscopy from blood plasma from HHT patients and a healthy control group. Spectral data were mathematically processed and subsequently classified and analysed by artificial neural network (ANN) analyses and by visual analysis of scatter plots of the dominant principal components.

**Results:**

The analyses showed that for HHT a disease specific IR-spectrum exists that is significantly different from the control group. Furthermore, at the current stage with the here used methods, HHT can be diagnosed by Mid-IR-spectroscopy in combination with ANN analysis with a sensitivity and specificity of at least 95%. Visual analysis of PCA scatter plots revealed an inter class variation of the HHT group.

**Conclusion:**

IR-spectroscopy in combination with ANN analysis can be considered to be a serious alternative diagnostic method compared to clinical and genetically based methods. Blood plasma is an ideal candidate for diagnostic purposes, it is inexpensive, easy to isolate and only minimal amounts are required. In addition, IR-spectroscopy measurement times are fast, less than one minute, and diagnosis is not based on interpretation of may be uncertain clinical data. And last but not least, the method is inexpensive.

## Background

The vascular disorder Hereditary Haemorrhagic Telangiectasia (HHT) affects 1 in 5.000 to 8.000 people and is in general inherited in an autosomal-dominant way [1,2], but de novo affections are also reported. The disease is thought to be present in all races and parts of the world [3]. HHT is commonly caused by mutations in either the endoglin [4] or ALK1 [5] gene and in rare cases in SMAD4. SMAD4 mutations lead to the combined syndrome of juvenile polyposis (JP) and HHT (JP-HHT) [6,7]. All three genes code for proteins involved in the TGF-beta/BMP pathways. By linkage analysis, two additional regions on chromosome 5q31.3-5q32 [8] and 7p14 [9], respectively, were identified that might harbour HHT related genes, which are so far not identified and will only count for a small number of HHT cases.

HHT is a vascular disorder and the research of the last 20 years has shown that the TGF-ß/BMP receptors endoglin and ALK1 and Smad4, as an intracellular signal transducer protein, are involved in the different angiogenic processes. On the micro anatomic level HHT is characterised by a distorted and convoluted capillary bed with shunts between the arterial and venous system. The mutations in the different HHT genes can lead to different phenotypes and clinical symptoms, which are a) spontaneous and recurrent epistaxis, b) multiple telangiectases at characteristic sites like lips, oral cavity, fingers and nose, and c) visceral lesions like gastrointestinal telangiectasia, pulmonary, hepatic, cerebral or spinal arteriovenous malformations [3]. The disease shows an inter- and intra-family variability in penetrance as well as symptoms from mild to life threatening and penetrance is also depending on age. Not all typical symptoms may be present, especially at a younger age, when a suspected HHT patient is presented to a physician. For example, several studies have reported an average age of 12 years for the onset for epistaxis, with nearly 100% affected by the age of 40 (reviewed in [2,3,10]. Furthermore, most patients report the appearance of telangiectasia of the mouth, face or hands 5 to 30 years after the onset of nose bleeds, most commonly during the third decade. In general, most patients show a full penetrance of the disease around the age of 40. However, in rarer cases the disease can start at an older age or very rarely the symptoms are that mild that these patients might be missed to have HHT. In the year 2000, consensus clinical diagnostic criteria, known as the Curacao Criteria, were established for the diagnosis of HHT [11]. These criteria include, aside from the clinical symptoms, also as a single independent criteria if someone has first degree relatives with HHT. By these guidelines, HHT is considered to be definite if three or more criteria are present, possible or suspected diagnosis if two criteria are present, and unlikely if none or only one criterion is present. Nevertheless, the penetrance of symptoms varies, also in regard of age, and therefore HHT patients might not be diagnosed with HHT. A recent questionnaire based study reports a long diagnostic delay in patients with HHT [12]. In this study the average clinical onset of disease occurred at an age of about 14 years. Whereas the age of first referral and the first definite diagnosis were at an age around 29 and 40 years, respectively. When a person is diagnosed, it is not unusual to identify multiple relatives in the family who have not been labelled with HHT but who have struggled for example with epistaxis, embolic stroke, or GI bleeding.

Based on the different clinical symptoms, HHT diagnosis can be laborious, time consuming, costly and uncertain. Aside of clinical diagnosis by different methods, genetic testing for the disease genes endoglin, ALK1 or Smad4 is a further but also costly option and the disease causing mutation might not always be detected. Therefore, it would be desirable to have a diagnostic tool at hand that facilitates diagnosis, especially for those physicians and general practitioners that have no experience with the disease.

A diagnostic tool that will lead to an earlier, faster, cheaper and easier diagnosis might be the infrared (IR)-spectroscopy. In the past years, Near- and Mid-IR spectroscopy, which is well established for quality control in the chemical industry and material sciences, has emerged as a possible alternative diagnostic method for different diseases and other purposes. IR-spectroscopy of body fluids like blood plasma, serum or urine has been shown to reveal disease-specific changes in spectral signatures, for in example bovine spongiform encephalopathy [13], diabetes mellitus [14], rheumatoid arthritis [15], β-Thalassemia [16] or myocardial infarction and heart failure [17]. Furthermore, IR-spectroscopy can also be used for measuring the concentration of different clinically relevant constituents of body fluids: albumin, cholesterol, glucose, total protein, urea, tri-glycerides, uric acid, phosphate, creatinine [18] or plasma haemoglobin [19]. IR-spectroscopy is based on the detection of molecular vibrations in different chemical groups of i.e. proteins, lipids, nucleic acids. The idea behind using IR-spectroscopy in diagnostic is that the disease leads to an overall change in molecule and group composition of the different molecules that is different from the spectrum of non-affected people, resulting in a disease-specific IR-fingerprint.

In this study, we used blood plasma in combination with Fourier-transform Mid-infrared spectroscopy and Artificial Neural Network (ANN) analyses in order to test if for HHT a disease specific IR-fingerprint exists and whether IR-spectroscopy from patient plasma might be a feasible diagnostic alternative to the current practice or not. Blood plasma is an ideal candidate for diagnostic purposes because it is inexpensive and easy to isolate. Our analyses showed that at the current stage we can diagnose HHT by IR-spectroscopy and ANN analysis with a specificity and sensitivity of at least 95%.

## Methods

### Patient and control samples

For this study, EDTA blood samples of 2.5 ml were sent to us either by the HHT patients or their attending physicians by regular postal mail. Transport time of samples was between one and three days but in general only one day. After blood sample arrival, 0,5 ml blood was stored away at -80°C for those patients with unknown genetic status, for possible future mutation analysis. From the remaining 2 ml, plasma was collected by centrifugation for 10 min at 4°C with at least 13.000 rcf. Plasma was stored at -20°C until use. The majority of patient samples were from German patients. However, a small number of 19 Italian patients and 4 Spanish patients plus one negative control were also included. The Italian samples were sent as plasma on dry ice, whereas the Spanish samples were sent as EDTA blood. Blood plasma from blood donors from the blood donating centre in Mannheim served as the healthy control group. These plasma samples were processed by the centre. The study was approved by the medical ethic committee II of the Medical Faculty Mannheim, University of Heidelberg. In order to decide upon the disease status of the patients they had to fill out a questionnaire regarding their symptoms, genetic status and whether first degree relatives are affected by HHT. Based on this information, only the spectra of patients that fulfilled three criteria for HHT were used for the spectral pattern analysis. Unfortunately, not for all patients the questionnaire was returned and their samples were excluded from this study.

### FT-IR spectroscopy

1 μl blood plasma was diluted in 3 μl HPLC-water and of this three times 1 μl were spotted randomly onto a 384 well Si-sample carrier at three different places. The plate was then dried overnight in a descicator. Next, the plate was placed into an HTS-XT micro plate reader and the spectra were recorded with a Tensor 37 FT-IR Spectrometer (Bruker Optics GmbH, Ettlingen, Germany). Mid-infrared spectra were measured by diffuse reflectance and were recorded in the spectral range between 4.000 and 400 cm^-1^.

### Data analysis of IR-spectra with Pattern Expert

Preliminary data processing was performed with the Optics User Software (OPUS) from Bruker Optics. In brief, the obtained spectra were preprocessed by vector normalization and the first and second derivative of the original spectra were formed using the Savitzky-Golay algorithm with nine smoothing points. Subsequently, the data was loaded into the Artificial Neural Network (ANN) software Pattern Expert Airspect (Pattern Expert, Borsdorf, Germany). For spectra and data analysis and configuration the programs standard settings for feature computation (ROI, regions of interest), feature selection (all features) and classifier construction (ANN linear) were used.

The samples were separated into two groups, healthy and HHT positive, based on our information from the answered questionnaires and the known mutations. The program then compares the IR bands of all healthy samples and all diseased samples to establish an average IR pattern for each respective group. Next, the average of each group is compared against one another to determine ‘regions of interest’ (ROIs), that is, regions with significant differences in the bands, which are used to decide whether a specific IR band belongs to the healthy or HHT positive group.

The next step is the supervised classification using an ANN. This step represents the learning phase of the automated neural network leading to a recognition module by which the spectra are classified into an HHT group and a healthy non-HHT group, summarized in a Classification Table (also denoted as confusion matrix). This table contains the results of a final stratified cross validation using the best recognition module found. Its rows correspond to the target class, i.e. the classes the spectra actually belong to, while the columns correspond to the ANN output class, i.e. those classes they have been assigned to by the recognition module. The diagonal elements of the table correspond to correctly assigned samples while the off diagonal elements correspond to false classification. Once the classification step is done the program performs a so called external cross validation. For every set of parameters used the program re-evaluates to which group each IR signal belongs to. After trying all combinations and discovering which set yields the most accurate predictions, an external cross validation is performed. This procedure involves randomly selecting 10% of the samples being used from each group and treating them as if they are unknown. They are then compared to the average signal of the remaining 90% of each group and assigned to the group their signal matches more exactly. This is repeated 10 times for each signal, using different ROIs each time. The ROIs used for this calculation come from the previously decided upon range. The multiple repetitions of each comparison lead to the program deciding upon a confidence level for each of its decisions, ranging from 50-100%. After the program has designated each of the selected spectra to a group it selects another 10% of the total samples and begins to evaluate them in the same way it studied the previous group. This process repeats itself until all 100% of samples being considered have been measured. After the cross validation is completed the samples can be reviewed individually to check the accuracy of each prediction and the programs confidence level in its decision

### Sequencing

DNA from blood samples (patients) 306 (ENG exon 11 mutation), 334 and the mother (ALK1 exon 9 mutation) of patient 334 were isolated with a DNA extraction Kit (Q-bios GmbH). Extracted DNA was used in a PCR for exon amplification. Exons were amplified in a standard PCR with a HotStart Taq Polymerase (Q-bios). Amplified exons were gel purified (Gel Purification Kit, Q-bios) and the purified exon DNA was used in a sequencing reaction with the Big Dye Terminator v1.1 Kit according to the manufacturer's instructions. Sequences were run on an ABI 310 Prism Genetic Analyzer (Applied Biosystems). For exon amplification the following primers were used: ENG exon 11 forward, gctaccacgagctcggccgatatttgaaggcagcag; ENG exon 11 reverse, 5'-gtcgccgtcgctctaggcaaaccacagacctggaag-3'; ALK1 exon 9 forward, 5'-gctaccacgagctcggggcctccttagagtcccaag-3'; ALK1 exon 9 reverse, 5'-gctaccacgagctcggcagaaatcccagccgtgag-3'; forward sequencing primer 5'-gctaccacgagctcgg-3'; reverse sequencing primer, 5'-gctaccacgagctcgg-3'. The underlined sequences in the exon primers are not exon-specific but are specific for the sequencing primers.

## Results

Most of the here used samples were analysed in two independent studies by two different investigators with similar results. In pre-emption of the results, both investigations showed that there is an HHT-specific IR-fingerprint. Here we present the results of the second study, which included more samples. In this study, 192 blood plasma samples from 191 HHT patients were analysed. The majority of samples, 169, were collected from 168 German patients with the help of the German patient group Morbus Osler Selbsthilfe e.V. Smaller sample numbers were provided from Italy (19) and Spain (4). The blood plasma from 191 blood donors served as the non-HHT control group. Plasma samples from 7 HHT relatives having no HHT (samples 162, 214, 214a, 306, 320, 332, 334, 354, 359, one sample from an Angioma serpiginosum patient (sample 310) and one non-HHT sample (sample 358) from Spain were added to the control group. In total, 202 samples. The average age of the HHT group was 54.1 years (+/- 12,6 years). The average age of the control group was 43.2 years (+/- 13,6 years). For two non-HHT persons from the HHT relative group we received two samples at different times, samples 214(214a)/354, and samples 306/359. Sample 214 and 214a represent the same blood probe but plasma from this probe was measured twice at different times. From the non-HHT group two people, corresponding to samples 334 and 306/359, were investigated for a mutation in ALK1 or endoglin, respectively. Sequencing confirmed that they do not carry the family-specific HHT mutation.

### Mutation status of analysed patients

For our first evaluation, in order to see whether an HHT-specific IR-spectrum exists or not, we only used samples from patients with a confirmed mutation in endoglin or ALK1. This group consisted of 63 patients. Seven more patient samples with a defined genetic status for endoglin, ALK1 or SMAD4 were included in the second analysis with a larger number of samples. In the German patient group 16 patients have an endoglin mutation, 29 an ALK1 mutation and two a SMAD4 mutation. For the Italian and Spanish group 7 endoglin and 16 ALK1 mutations were reported. The mutations are listed in Table 1. The majority of mutations are already known and are listed in the HHT Mutation Database (http://www.arup.utah.edu/database) hosted by the University of Utah Department of Pathology and ARUP Laboratories. However, 17 mutations were not found in the database and might be new.

**Table 1:** List of mutations of genetically confirmed HHT patients

**Patient (sample) number**	**Patient group**	**Mutation**	**Reported in ARUP data base / not reported in data base**	**Affected organs/clinical symptoms**	**Age (in years)**
74	German	ALK1 Exon 9, c.1297C > T (p.P433S)	HHT data base	no information	54
105	Spanish	ALK1 Exon 8, c.1205 G > A, p.G402D	HHT data base	no information	30
106	Spanish	ALK1 Exon 8, c.1205 G > A, p.G402D	HHT data base	no information	64
165	German	ENG (c.1712delG)/(p.Arg571fr)	HHT data base	E, T	40
166	German	ENG, c.324-325InsT (Codon 109)	HHT data base	E, T, PAVM, HAVM, GI, PH	66
169	German	ALK1, p.Q292P (Exon 7)	HHT data base	E, T	69
178	German	ALK1, c926-930del, GGCCATCAGGAAAA (IVS5-8)	not reported	E, T, PAVM, GI	69
182	German	ALK1 (c.313 + 1_313 + 13delGTACGTCCAGCTG)(p.L106fsX129)	not reported	E, T	64
183	German	ALK1, c.905T > G, p. L302R	HHT data base	E, T, GI	67
184	German	ALK1, c221delG, pR74fx47	not reported	T, GI	80
199	German	ENG, c.1015-1024delGCACCGATCC (Exon 8)	not reported	E, T, PAVM	61
201	German	ALK1, c.186-190delCCCC (Exon 3)	not reported	E, T	81
202	German	ALK1, c.570-573delCCCC (Exon 6)	not reported	E, T, HAVM, bladder	81
204	German	ENG, c.1243C-T, p.Q415X	HHT data base	E, T, PAVM	66
208	German	ALK1, c.806C-A, p.S269X	not reported	E, T	54
216	German	ALK1, c.1207C > G, p.L403V	not reported	E, T, HAVM	60
223	German	ENG, c.360G > A Intron 3	HHT data base	E, T	69
228	German	ALK1, c.881T > G, p.L294R	not reported	E, T, PAVM	39
232	German	ALK1 gene deletion (one allele)	not reported	(E), T, PAVM	51
241	German	ALK1, c.1130C > A, p.A377E, Exon 8	not reported	E, T, GI	59
254	German	ENG, c.1015-1024delGCACCGATCC (Exon 8)	not reported	E, T	56
258	German	ALK1, c.1048G > A, p.G350S	HHT data base	E, T, CAVM, HAVM, GI	61
259	German	ALK1, c.905T > G, p. L302R	HHT data base	E, T	67
264	German	ALK1 (c.313 + 1_313 + 13delGTACGTCCAGCTG)(p.L106fsX129)	not reported	no symptoms reported	36
272	German	ENG, c.274-277delC (Exon 3)	not reported	E, T, PAVM, HAVM	69
279	German	ALK1, c.998G > T, p.S333I (Exon 7)	HHT data base	E, T, HAVM	74
280	German	ENG, c.1686 + 1G > A (Splice Mutation)	not reported	E, T, CAVM, PAVM	41
283	German	ENG, c.324-325InsT, Codon 109	HHT data base	E, T, PAVM	68
284	German	ALK1, c.1120 C > T, p.R374W	HHT data base	no information	64
292	German	SMAD4, c.1087T > C; p.C363R	Smad4 data base	E, T, PAVM, GI, JP	35
297	German	ENG, c.1103T > C, p.M368T	HHT data base	E, T, PAVM	45
298	German	ENG, c.1103T > C, p.M368T	HHT data base	E, T	67
299	German	ENG exon 11, c.1432-33 delAGfs	HHT data base	E, T, PAVM, PH	71
301	German	ALK1 Exon 3, c.200G > A, p.R67G	HHT data base	E, T	65
304	German	ALK1, c.998G > T, p.S333I (Exon 7)	HHT data base	E, T, HAVM	49
316	German	ENG, c.duplikation Exon2-4	HHT data base	E, T, PAVM, GI	57
317	German	ENG, c.360 + 5G > T (IVS3 + 5G > T)	not reported	E, T, PAVM, GI	68
324	German	ENG, c.360C > A (Exon 3), p.Y120X	HHT data base	E, T	60
325	German	SMAD4, c.1157G > A, p.G386D	Smad4 data base	E, T, PAVM, JP	39
326	German	ALK1 gene deletion (one allele)	not reported	(E)	23
327	German	ALK1, c.145-146InsG (GCC49GGCC)	HHT data base	E, T, GI	78
329	German	ALK1 Exon 5, c.540-541insA	HHT data base	E, T	39
330	German	ENG Intron 3, c.361-2A > G	HHT data base	E, T, PAVM, spinal abscess, stroke	44
333	German	ALK1, Exon8, c.1231G > A, p.R411Q	HHT data base	E, T	67
339	German	ALK1, Exon 5, c.540-541insA	HHT data base	(E), T	39
342	German	ALK1 Exon 5, c.540-541insA	HHT data base	E, T	42
347	German	ALK1, Exon 7, c.788A > G, p.Asp263Gly	not reported	E, T, PH	37
353	German	ALK1 (c.1120 C > T)(p.Arg374Trp)	HHT data base	E, T	45
356	Spanish	ENG, exon 2, pF71fs	HHT data base	no information	-
357	Spanish	ALK1, exon 7, pH297fs	not reported	no information	-
360	Italian	ALK1, Exon 3, c.289_294 delCACAAC (p.H97_N98del)	HHT data base	no information	27
361	Italian	ALK1, Exon 8, c.1112 G > A (p.G371D)	not reported	no information	66
362	Italian	ENG , Exon 8, c.1097_1119 del23bp (p.D365EfsX22	HHT data base	no information	3
363	Italian	ALK1, Exon 8, c.1231 C > T (p.R411W)	HHT data base	no information	25
364	Italian	ALK1, Exon 7, c.824 dupGGCT (p.L275LfsX118)	HHT data base	no information	40
365	Italian	ALK1, Exon 4, c.314-3 C > G (Splice Site)	HHT data base	no information	37
366	Italian	ENG, Exon 3, c.360 + 1 G > A (Splice Site)	HHT data base	no information	65
367	Italian	ALK1 , Exon 6, c.743_744 delCA (p.T248SfsX142)	not reported	no information	63
368	Italian	ALK1, Exon 7, c.809_821 delCACGCAGCTGTGG (p.270SfsX27)	HHT data base	no information	72
369	Italian	ENG , Exon 8, c.1085_1086 insA (p.T361fsX395)	not reported	no information	68
370	Italian	ALK1, Exon 7, c.853 dupC (p.L285PfsX107)	not reported	no information	66
371	Italian	ALK1, Exon 10, c.1435 C > T (p.R479X)	HHT data base	no information	44
372	Italian	ENG, Exon 3, c.360 + 1 G > A (Splice Site)	HHT data base	no information	77
373	Italian	ENG, Exon 3, c.277 C > T (p.R93X)	HHT data base	no information	73
374	Italian	ALK1, Exon 3, c.200 G > A, p.R67G	HHT data base	no information	48
375	Italian	ENG, Exon6, c.816 + 5 G > C (Splice Site)	HHT data base	no information	38
376	Italian	ALK1, Exon 8, c.1232 G > A (p.R411Q)	HHT data base	no information	49
377	Italian	ALK1, Exon 8, c.1231 C > T (p.R411W)	HHT data base	no information	64
378	Italian	ALK1, Exon 7, c.809_821 delCACGCAGCTGTGG (p.270SfsX27)	HHT data base	no information	63
379	German	ALK1, Exon 3, c.286A > G; p.N96D	HHT data base	T, PAVM	23

*AVM* arterio-venous malformation, *E* epistaxis every two weeks or more or daily episodes; (E), on average epistaxis once a month or less; *T* telangiectases (lips, oral cavity, fingers and nose), *CAVM* cerebral AVM, *HAVM* hepatic AVM, *PAVM* pulmonary AVM, *GI* gastro intestinal telangiectases, *PH* pulmonary hypertension.

### Analysis of spectra from genetically defined patients with the Pattern Expert Airspect software

**Figure 1:**
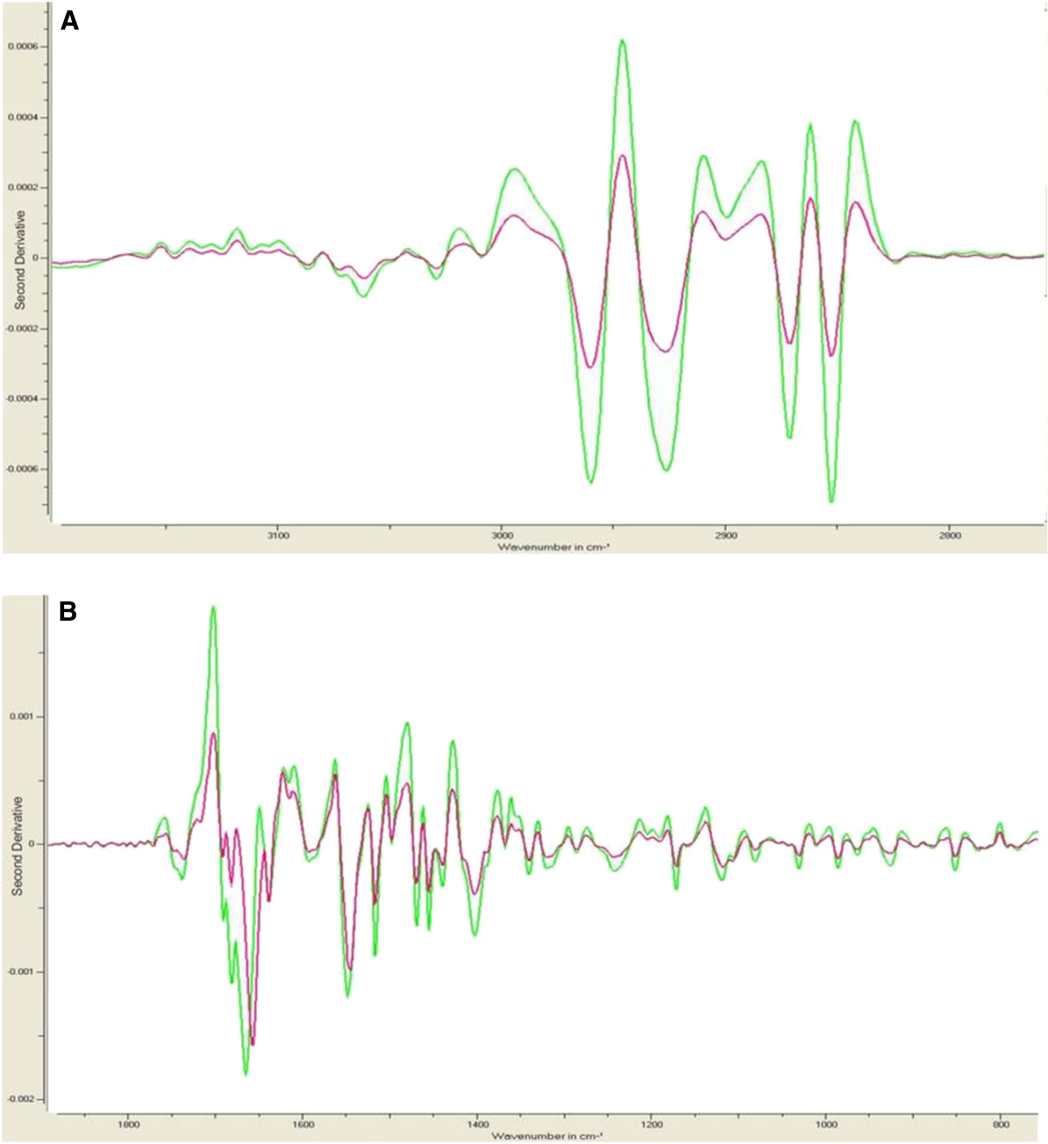
**Second derivative spectra of healthy control and genetically confirmed HHT patients.** Shown is the mean spectrum of all 191 control spectra in green and the mean spectrum of 63 patient spectra in red for spectral regions 3150–2750 cm^-1^ (**A**) and 1.850 - 750 cm^-1^ (**B**).

In order to evaluate whether HHT patients show a disease-specific fingerprint in the Mid-IR spectrum or not, we first tested our hypothesis with 63 genetically confirmed endoglin and ALK1 HHT patients. This analysis clearly showed that the spectrum of HHT patients is different from the control group of 191 blood donors. For the evaluation of the spectra with an Artificial Neural Network (ANN) the Pattern Expert Airspect software was used. The spectra were analysed with the programs standard setting. For the analysis two spectral regions from 3150–2750 cm^-1^ and 1.850 - 750 cm^-1^ were selected. Therefore, excluding a possible water band at 3350 cm^-1^ and the CO_2_ band at 2200 cm^-1^. Figure 1 shows the mean second derivative spectra for all patients (red line) and all controls (green line).

By simple visual comparison a difference between the two spectra can already be observed. The control group waves show higher amplitude at different wave number positions than the HHT group but follow the same wave pattern except for the region from 1750 cm^-1^ to 1600 cm^-1^. After the learning phase, 190 blood donors of 191 were correctly classified into the non-HHT group (Table 2). Of the 63 patients, 60 were classified into the HHT group. Next, the program performed the external cross validation, which can be considered as the training and validation/test phase in one step. Based on this, blood donor samples 84_Av and 11_Av were classified into the HHT group with a very high and a very low confidence level (see Table 3), respectively. After cross validation, five HHT patients were miss-classified to belong to the “healthy” blood donor group with high confidence levels for samples 333, 342, 371 and low confidence levels for samples 254 and 297 (see Table 3). There might be a high uncertainty regarding a correct classification for samples with confidence levels of around 50%, because these samples are close to the decision boundary with most likely a high standard deviation, an output the program unfortunately does not show.

**Table 2:** Classification table for the genetically confirmed HHT patients and the healthy control group

	**Assigned negative**	**Assigned positive**	**Sum**
**Negative (blood donors)**	190 (99,5%)	1 (0,5%)	**191**
**Positive (genetically confirmed HHT patients)**	3 (4,8%)	60 (95,2%)	**63**
**Sum**	**193**	**61**	**254**

Classification is based on a recognition module established by the Pattern Expert Airspect program after analysis of both spectral regions 3.150 – 2.750 cm^-1^ and 1.850 - 750 cm^-1^.

**Table 3:** Confidence level of misclassified genetically confirmed HHT patients after analysis of both spectral regions

**Non-HHT samples classified into the HHT group (false positive)**	**HHT patient samples classified into the control group (false negative)**
15_Av (52%)	254 (56%)
84_Av (99%)	297 (59%)
	333 (100%)
	342 (74%)
	371 (85%)

Confidence levels were calculated by the Pattern Expert Airspect program after ANN and cross validation analysis with blood donor samples and genetically confirmed HHT patient samples for spectral regions 3.150 - 2.750 cm^-1^ and 1.850 - 750 cm^-1^.

For the evaluation, the ANN was set to analyse up to 300 spectral features spread over two spectral regions but we do not know how many features the program really used. Sometimes, one spectral region is less informative than the other and thus, one spectral region may be neglected for analysis. The spectral difference, which we observed between 1750 cm^-1^ and 1600 cm^-1^, suggested that the region 1.850 - 750 cm^-1^ is more informative and therefore, sufficient for an ANN analysis and might lead to an improved prediction. This, however, was not the case. After cross validation, only blood donor sample 84_Av was still classified into the HHT group with about the same high confidence level as before. Again, five patient samples were classified to belong to the control group. But it is interesting and important to note that this time samples 254 and 371 were not in the false negative group anymore but were replaced by 199 (55% confidence) and 327 (68% confidence). Furthermore, the confidence levels for samples 297 and 342 increased to 72% and 96%, respectively. These first data show that there is an HHT disease specific IR-fingerprint and that both analysed spectral regions contain HHT-specific important features but with a higher weighting for features in region 1.850 - 750 cm^-1^. Based on the results from the external cross validation (Table 4) with this relatively small group of patients we calculated a sensitivity of 92% [correct positive/(correct positive + false negative)] and a specificity of 99% [correct negative/(correct negative + false positive)].

**Table 4:** Classification of all HHT patients and the healthy control group after spectra analysis

	**Assigned negative**	**Assigned positive**	**Sum**
**Negative (control group)**	193 (95,5%)	9 (4,5%)	202
**Positive (HHT patients)**	8 (4,2%)	184 (95,8%)	192
**Sum**	201	193	394

Classification is based on a recognition module established by the Pattern Expert Airspect program after analysis of both spectral regions 3.150 - 2.750 cm^-1^ and 1.850 - 750 cm^-1^.

### Analysis of spectra from all HHT patients with the Pattern Expert Airspect software

The previous experiment and analysis has proven that for HHT a disease-specific fingerprint in the Mid-IR spectrum exists. This fingerprint was established with a relatively small number of samples. Thus, in the next step, we repeated our analyses with a larger number of HHT patients, in total 191, including all genetically confirmed patients and those patients that fulfilled the Curaçao criteria, represented by 192 plasma samples. For one patient we had two samples collected at different times. The control group consisted of plasma samples from the blood donors as well as a group of non-HHT affected HHT relatives, in total 201 samples.

**Figure 2:**
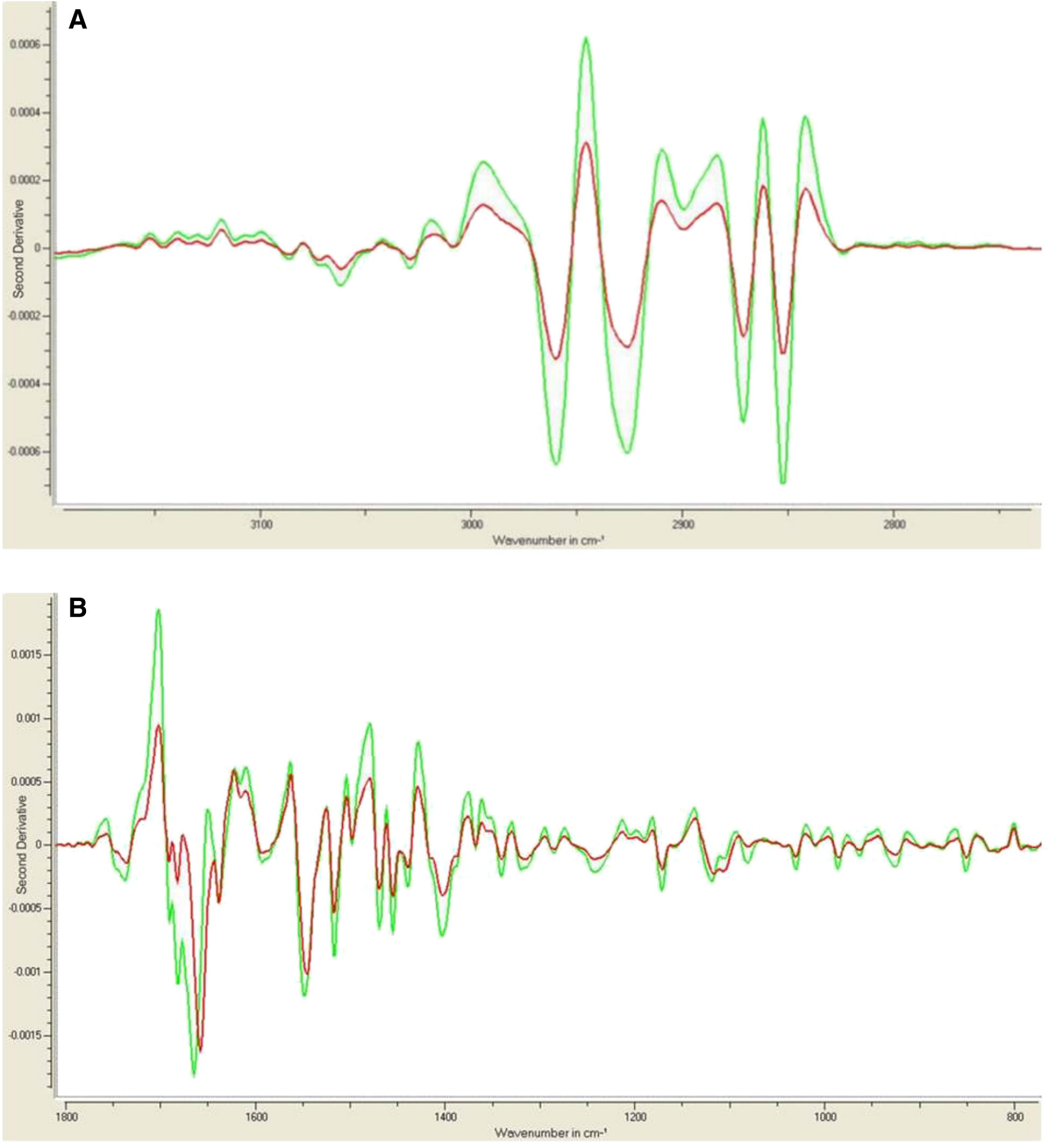
**Second derivative spectra of healthy control and all HHT patients.** Shown is the mean spectrum of 202 control spectra in green and the mean spectrum of 192 patient spectra in red for spectral regions 3150–2750 cm^-1^ (**A**) and 1.850 - 750 cm^-1^ (**B**).

Figure 2 shows the mean second derivative IR-spectrum of the healthy control group (green line) and the HHT group (red line). In Table 4 the classification results are summarized. In Table 5 those samples are shown that were possibly misclassified after the external cross validation analysis. Samples are listed with their confidence level. The classification results gave a specificity and sensitivity of about 96% but based on the results from the external cross validation (Table 5) we calculated after the analysis of this larger group a sensitivity and specificity of 95%.

**Table 5:** Confidence level of misclassified HHT patients after analysis of both spectral regions

**Non-HHT samples classified into the HHT group (false positive)**	**HHT patient samples classified into the control group (false negative)**
3_Av (65%)	287 (78%)
15_Av (73%)	297 (80%)
84_Av (99%)	300 (84%)
95_Av (74%)	324 (57%)
162 (99%)	325 (70%)
214 (78%)	330 (83,5%)
214a (69%)	333 (100%)
306 (82,5%)	348 (99%)
334 (88%)	350 (93%)
358 (100%)	356 (97%)

Confidence levels were calculated by the Pattern Expert Airspect program after ANN and cross validation analysis with all HHT samples and all control samples for spectral regions 3.150 - 2.750 cm^-1^ and 1.850 - 750 cm^-1^.

Like before, we repeated the evaluation by analysing only the spectra for region 1.850 cm^-1^ – 750 cm^-1^. Again, no major difference or improvement in sensitivity and specificity of disease prediction was observed. The group of false positives and negatives consisted mostly of the same samples as before. The group of false positives were the same except for samples 3_Av and 95_Av, which were assigned to the negative control group. The group of false negatives again were sample 300 (96%), 330 (87%), 333 (100%), 348 (86,5%), 350 (93%) and 356 (100%), plus samples 352 (57%), 353 (53%) and 199 (50%) but this time without samples 287, 297, 324, 325. The numbers in brackets represent the confidence level. The classification of samples 199, 352 and 353 has to be interpreted with caution because of the low confidence level. Again, there is a high uncertainty regarding a correct classification for samples with confidence levels of around 50% as explained before. These data suggest that under the analysis parameters chosen here both spectral ranges contain disease specific regions and therefore should be included in the ANN analysis for an optimal prediction.

### Bhattacharyya coefficient based feature selection and ANN analysis

This first analysis of the spectra by a commercial customer friendly ANN analysis software package provided already very good results. But we were concerned regarding some of the false positive and negative calls. Especially in those cases where we knew that these patients are definitely HHT positive or HHT negative. Therefore, we started to reanalyse the spectra in more detail, which can also be considered to be the more “classical” approach.

**Figure 3:**
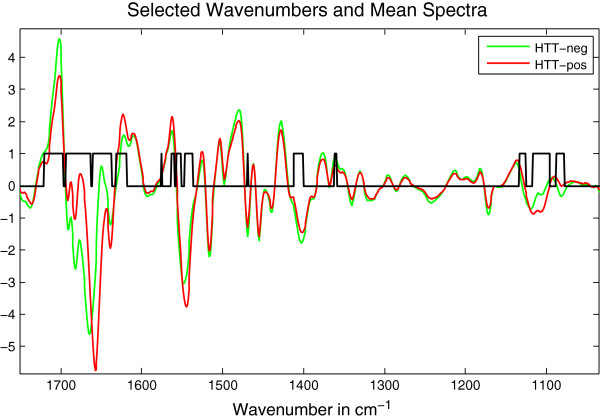
**Relevant wavenumbers/features used for ANN analysis of HHT patient and control group spectra.** For each wave number the Bhattacharyya coefficient was calculated. A wavenumber was selected if Bhattacharyya coefficient was greater than 5% of the greatest coefficient. The selected wavenumber regions are marked by the black lined peaks and open rectangles.

For this analysis, again the regions 3150 cm^-1^ - 2750 cm^-1^ and 1850 cm^-1^ - 750 cm^-1^ were selected from the pre-processed spectra and both regions were normalized separately to zero mean and variance 1. Next, we performed the feature selection. In order to identify those wave numbers (features) that allow for the best differentiation between patient and healthy control group (classes), we used the Bhattacharyya coefficient. For each wave number the Bhattacharyya coefficient was calculated. A wave number was selected if Bhattacharyya coefficient was greater than 5% of the greatest coefficient. By this criterium 238 features distributed over both regions were identified to be relevant in order to differentiate between the non-HHT and HHT group. However, at high wavenumbers, 3050 cm^-1^ - 2750 cm^-1^, the relevant wavenumber regions are sparse and have only low Bhattacharyya coefficients between 5% - 9%. Thus, Figure 3 shows only the selected relevant features for region 1850 cm^-1^ - 750 cm^-1^.

The Bhattacharyya coefficient in this application was superior to t-test, entropy, roc or Wilcoxon (Mann-Witney U test) feature ranking concerning misclassification. Once the relevant features in order to differentiate between HHT and non-HHT were defined, a 1-hidden layer feed forward neural network with 3 hidden layer neurons was trained 67 times using the spectral data of the HHT and non-HHT samples. In each session the neural network was trained with randomly selected 70% of the total number of samples. Of the remaining 30%, half of it, again randomly selected, was used for training validation and the other half (test data, representing 15% of the total) was used for testing the networks training success. Only the test data were used to calculate the performance. The validation data were used for early stopping in order to avoid over training. This process (training and testing) was repeated 67 times. As a consequence, each observation (spectrum) was used in ten different test data sets (0.15 × 67 = 10.05). Because the test data are not used in the training this provides a completely independent measure of the network accuracy.

**Figure 4:**
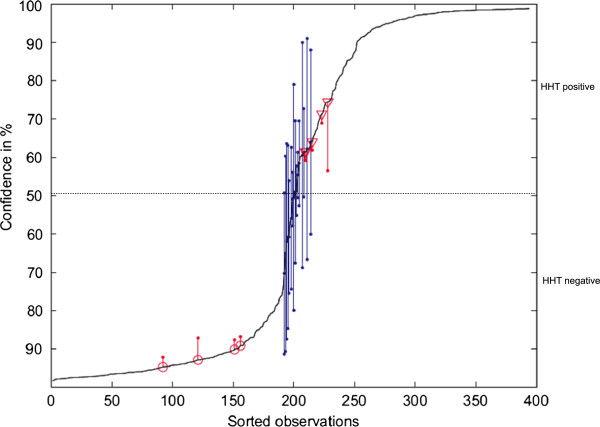
**Confidence levels of ANN based classification.** The measure of confidence of the ANN (soft decision output) is a number between 0 and 1. 0.5 is interpreted as 50% confidence, 0 and 1 as 100% true negative or 100% true positive, respectively. Based on the ANN soft decision output the spectra are placed on the curve, corresponding to their confidence level. The term “Sorted Observations” refers to the number of analysed spectra. Each observation represents one spectrum. False negative spectra are marked by a circle (with standard deviation, red line) and the false positive spectra are marked by a triangle (with standard deviation, red line). The blue lines mark the undefined spectra whose standard deviation crosses the 50% confidence decision boundary.

In order to get a measure of the reliability the soft decision network output is stored for each training session and for each observation the mean and the standard deviation is calculated. By this, a classification was considered to be reliable if the distance between the mean and the decision level 0.5 is greater the standard deviation. All other observations were labelled as undefined. The soft decision output of the NN is a number between 0 and 1. The soft decision output is a measure of the confidence: 0.5 is interpreted as 50% confidence, 0 and 1 as 100% true negative or 100% true positive, respectively (see Figure 4).

After 67 sessions of neural network training 6 samples, out of 394, were reliably classified as false positive (triangles in Figure 4) and 4 samples as false negative (circles in Figure 4) (see Table 6). Their standard deviation (calculated from at least 10 test data sets) did not reached or crossed the decision point of 0.5 (50% confidence). Based on our analysis criteria 21 samples (5%) could not reliably be classified (see Table 6, undefined group). Their standard deviation crossed the decision point as shown in Figure 4, represented by the blue lines. In the sessions, typically 96% +/-1% of the test data were classified correctly with similar sensitivity and specificity. But more importantly for future investigations, which sample was wrongly classified differed considerably depending on what data were selected for training and validation. By excluding the undefined sample group we calculated a sensitivity of 98% +/- 0.5% and a specificity of 97% +/- 0.5%.

**Table 6:** Summary of misclassified and undefined samples after ANN analysis using 238 spectral features

**Non-HHT samples classified into the HHT group (false positive)**	**HHT patient samples classsified into the control group (false negative)**	**Undefined samle group**
84_Av	333	3_Av
85_Av	348	5_Av
162	350	15_Av
214	352	22_Av
214a		37_Av
358		86_Av
		95_Av
		74
		204
		287
		300
		306
		325
		334
		336
		341
		342
		344
		351
		353

### Intra and inter class Principal Component Analyses (PCA) reveal overlapping and multi-modal clusters for the HHT-negative and -positive groups

Based on the afore performed ANN analysis, about 5% of the analysed spectra of the HHT group as well as the control group could not reliably be classified and 4 true HHT patients were misclassified. We wondered whether there might be a higher degree of spectral overlap between the two groups and also a higher spectral intra group heterogeneity as may be expected, i.e. that the two groups do not fall into two well separated clusters but overlapping clusters and that the clusters of each group show a multi modal characteristic.

**Figure 5:**
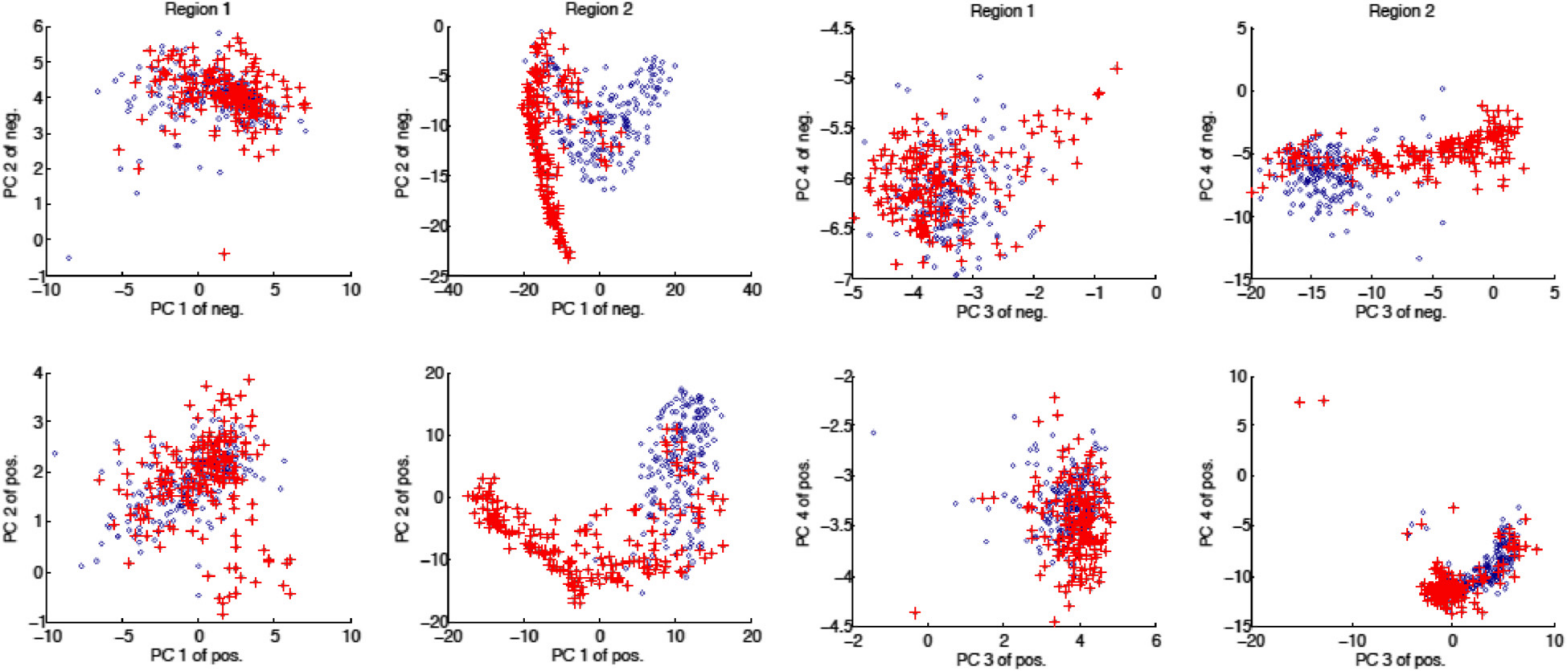
**Intra class principle component analysis (PCA) for the HHT-negative and HHT-positive group (class).** The four best eigenvectors for the HHT-negative (blue circles) and the HHT-positive (red crosses) group were seleted separately for region 1, 3150–2750 cm^-1^, and region 2, 1850–750 cm^-1^. Projecting the data onto the eight eigenvectors, resulting in 2x8 features (PC1-PC4 of the positive and negative group and of region 1 and 2), shows that the HHT-positive and HHT-negative groups are multimodal (multiple clusters) with an overlapping mode between the two classes.

First, we performed an intra class principle component analysis (PCA) in order to search for correlated observations within a class. For both spectral regions (region 1, 3150 cm^-1^ - 2750 cm^-1^; region 2, 1850 cm^-1^ - 750 cm^-1^) we selected the four strongest eigenvectors for the HHT-negative and HHT-positive groups (class) separately and projected the data onto the eight eigenvectors resulting in 2×8 features (PC1-PC4 of the positive and negative group and of region 1 and 2). The analysis revealed that the spectral region 2 is more selective (better cluster separation) than region 1 and both the HHT-positive and HHT-negative class are multimodal (multiple clusters) with an overlapping mode and in addition for both classes some outliers were observed (Figure 5). This confirms our hypothesis that there is to some degree an intra class spectral heterogeneity.

**Figure 6:**
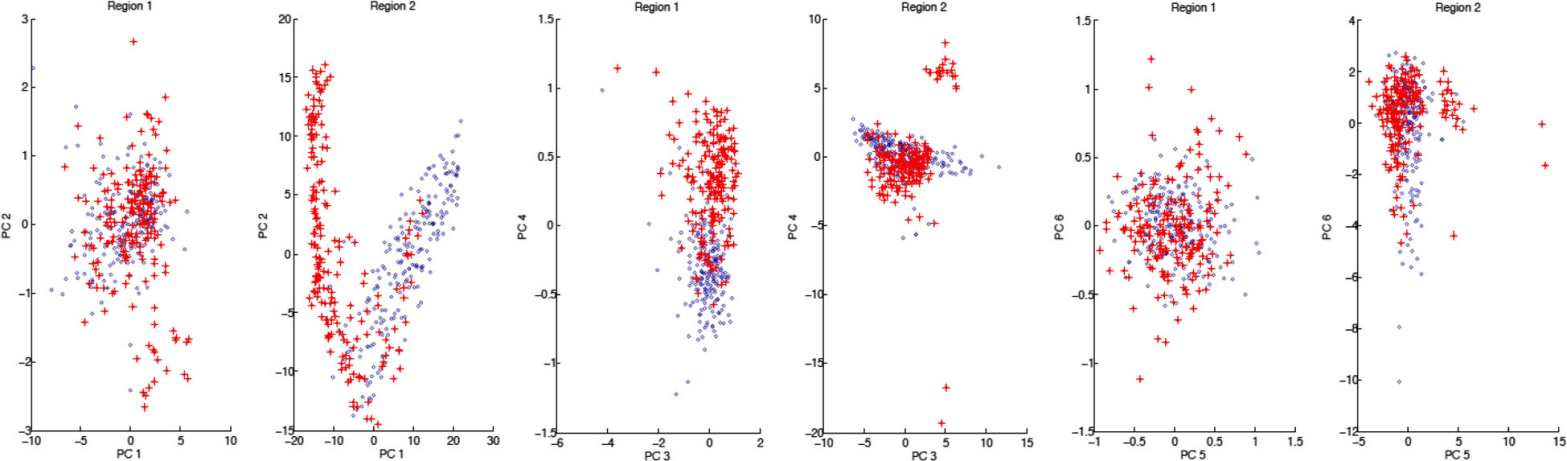
**Inter class principle component analysis (PCA) between the HHT-negative and HHT-positive groups.** The analysis was done for region 1, 3150–2750 cm^-1^, and region 2, 1850–750 cm^-1^. The projection shows that the spectra for the HHT-positive group (red crosses) and HHT-negative group (blue circles) are multimodal with overlapping modes and outliers.

Next, we did inter class PCA, i.e. we calculated the common eigenvectors of the HTT-negative and HTT-positive group and projected the data on these eigenvectors. Again, the spectra for the HHT-positive and HHT-negative group are multimodal with overlapping modes and strong outliers can be observed (Figure 6). These results might explain why the ANN had problems to classify some spectra correctly. Future investigations need to analyse whether these are regular biological variations or influenced by other factors like sample transport, processing or instrument dependent data recording.

## Discussion

The goal of this study was to evaluate if Mid-IR spectroscopy can be used for the diagnosis of HHT. Here, we have demonstrated now for the first time that the IR-spectra of HHT patients show a disease-specific IR-fingerprint and clearly differ from that of a healthy control group. Thus, IR-spectroscopy in combination with Artificial Neural Network (ANN) analysis can be considered to be a serious alternative diagnostic method compared to clinical and genetically based methods.

This pilot study by using two different ANN analyses yielded already a sensitivity and specificity of about 95% to 98% depending on, which method, analysis step and what data was used for the calculation. We are convinced that improvements and changes in ANN analysis parameters, increased sample numbers and sample preparation/transport will most likely lead to a further increased sensitivity and specificity. We have shown for the ANN analysis with the Pattern Expert program that concentrating on two or only one region can lead to slightly different predictions. In addition, the ANN analysis was done with the software programs standard settings. Our second ANN analysis approach has shown that the number of features, the number of training sessions and the selection of training and validation data sets influence the ANN decision. Furthermore, over the course of our study, we have seen that with increasing numbers of patient samples the prediction accuracy of the method improved. Future work has now to concentrate on the development of an improved algorithm by modifying program parameters, settings and data pre-processing.

It is important to note that the here presented method of IR-spectroscopy is not measuring the change of a single protein or biomarker but encompasses the overall metabolic change caused by a disease, which is then mirrored by a specific IR-spectrum or biochemical fingerprint in different body fluids. This can be clearly seen in Figure 3. The identified wavenumbers for the feature selection represent regions for biomolecules like proteins (~1800 cm^-1^ – 1400 cm^-1^) and DNA/RNA (~1400 cm^-1^ – 1000 cm^-1^) in general with may be specific peaks for lipids (~1750 cm^-1^), proteins secondary structure represented by the Amide I and II bands at ~1650 cm^-1^ and ~1550 cm^-1^ and carbohydrates/glycogen (~1155 cm^-1^/~1030 cm^-1^) [20]. The selected wavenumbers suggest that the majority of metabolic changes in HHT patients affect the biomolecule group of proteins.

It is possible that some patients, despite their disease and may be independent of their disease status, still show a metabolic “fingerprint” that is closer to the average spectrum of healthy people than to the spectrum of HHT patients and of course vice versa. As a simple example, some patients might have regular and sometimes dramatic episodes of nose bleeds but still normal hemoglobin values. The more samples of patients and healthy people are analysed by an ANN the more likely it is that the ANN learns to classify these “outliers” to the correct group. Therefore, we are convinced that more and more samples will lead to a higher sensitivity and specificity of this method. In line with this, our inter and intra class PCA analyses with all spectra demonstrated that both groups (classes), HHT and non-HHT, are not characterized by a single modality, but are multi modal. We would hypothesize that the inter and intra class observed spectral heterogeneity is more likely caused by a natural biological and physiological variance. In regard of this, it is interesting to note, that the spectra 214 and 214a represent the same sample, measured at different times but with the same result. This would suggest that instrument and measurement conditions can most likely be excluded as a factor for this false positive result.

Nevertheless, other factors like sample transport and plasma preparation cannot be excluded and might play an additional role. For example, there was less spectral heterogeneity among the blood donor group than the HHT group. The blood donor samples were provided to us by the blood donor centre as frozen plasma, prepared after a standardized protocol, whereas the HHT EDTA-blood samples were collected over a period of about 3 years and sent to us by regular postal mail. Once we received the EDTA-blood, the plasma was instantly prepared and stored away at -20°C. In general, transport time was one to two days by regular mail. However, we do not know how much time passed until the blood was sent to us and to what temperatures the blood was exposed to during transport. Interesting enough, two out of three samples from Spain, samples 356 (ENG mutation) and 358 (non-HHT control), that were sent to us by postal mail and took three days, were misclassified. But it is also possible that the samples were mislabelled. Then, the prediction would be correct. A sample from a healthy non-HHT person, sample 214, took several days before it reached us and was classified to belong to the HHT group. When we asked for a second probe about a year later this sample was correctly classified healthy. We wanted to keep the shipping for this study as simple as possible and overall our data show that this is a feasible way. However, for future studies it should be considered to process the blood as soon as possible. Furthermore, we recommend that once a person is classified to have HHT a second sample is ordered and measured. We had one case, sample 306, classified to have HHT but by sequencing we excluded the family specific mutation. When we analysed a second sample about a year later, this time the person was correctly classified to belong to the healthy group. What might have caused the first false prediction could have been a prolonged episode of a slipped disc at that time leading to inflammation like responses as the patient’s blood count suggests. It is well known that inflammation, especially for chronic inflammatory disorders, induces angiogenesis or angiogenesis like processes [21,22]. One of many features of these is an increased expression of the angiogenesis factor VEGF, which is also reported for HHT patients [23,24].

Other samples might be misclassified because the patients do not just have HHT but other diseases. For example, sample 325, which was classified into the false negative/undefined group, is a JP-HHT patient with a SMAD4 mutation. Obviously the patient’s spectral pattern does not exactly fit the HHT spectrum. By analysing a sufficient amount of JP-HHT patients it might be possible to differentiate between HHT and JP-HHT patients. Furthermore, other diseases may have symptoms that can cause an HHT-like IR-fingerprint. Such a case might be 334, the son of a genetically confirmed HHT mother, who was classified into the false positive or undefined group. By DNA sequencing, we excluded for the son the family specific mutation. However, on his questionnaire he stated to have an HAVM but otherwise no other HHT-typical symptoms. One proband, 162, the daughter of an HHT mother, was considered to have no HHT, based on her questionnaire, but her sample was consistently classified into the false positive group. She might be one of the rare cases that still do not show any symptoms at an age of 65 but her IR spectrum suggests that she might have HHT. For all the false positive, false negative and undefined cases it would be of course desirable to get a fresh sample for a second analysis.

By using IR-spectroscopy as a diagnostic tool, we do not think that IR spectra from other diseases will significantly compromise the HHT-specific IR-fingerprint leading to a high number of false positive classifications. For this purpose, the average spectrum of the HHT group was compared to a group of 99 prostate cancer samples and a group of 432 bronchial carcinoma samples. As a result, the HHT spectra were clearly differentiated from the prostate cancer and bronchial carcinoma spectra (data not shown). In addition, we tested whether gender might influence the analysis. There was no difference between female and male spectra. We also tried to differentiate between endoglin and ALK1 mutation carriers but no spectral difference was observed between these two groups.

Aside of demonstrating that IR-spectroscopy is a promising diagnostic method, we can also present several new endoglin and ALK1 mutations due to the information the patients provided from the questionnaires. Based on this, 16 german patients reported 11 different endoglin mutations, 29 german patients reported 22 different ALK1 mutations and 2 german patients a SMAD4 mutation. Previous mutation analyses for german HHT patients have shown that the endoglin ALK1 mutation ratio in Germany is slightly shifted towards ALK1 [25,26]. Our data do not only confirm this observation but suggest that ALK1 mutations are far more frequent in the german HHT population than endoglin mutations as it is also the case for the European-Mediterranean population.

## Conclusion

With the here used methods, HHT can be diagnosed by Mid-IR-spectroscopy in combination with ANN analysis with a sensitivity and specificity of at least 95%. Future work needs to concentrate on the aspects of sample transport and processing and increasing sample numbers to help to improve ANN analysis. Furthermore, in the current study most of the analysed probands showed typical HHT symptoms, with a few exceptions. Therefore, the question is, can we diagnose HHT by IR-spectroscopy at an early stage or young age when HHT symptoms are not yet present, easily missed or misinterpreted. Some of our results might suggest that this is possible. In order to answer this question, yet symptom-less members of HHT families need to be screened by IR-spectroscopy in combination with a genetic profiling for endoglin, ALK1 and SMAD4 mutations. An initially expensive investigation, but worthwhile for patient monitoring and surveillance that in addition might help to reduce costs for our health systems. The advantage of IR-spectroscopy in combination with ANN analysis is, once a disease-specific algorithm is established, no recalibration of the system is required. In addition, each new sample can be used to further train the ANN for an even more disease-specific algorithm. Further advantages are first, only minimal amounts of just 1 μl plasma are required. Second, measurement times are fast, less than one minute. Third, diagnosis is not based on interpretation of may be uncertain clinical data. And last but not least, the method is inexpensive.

## Abbreviations

ANN: Artificial neural network; BMP: Bone morphogenetic protein; FT: Fourier-transform; HHT: Hereditary hemorrhagic telangiectasia; IR: Infrared; JP: Juvenile polyposis; PCA: Principle component analysis; ROI: Region of interest; TGF-β: Transforming growth factor-β.

## Competing interests

The authors declare that they have no competing interests.

## Authors’ contributions

AL - Designed the project, co-ordinated the study, did the patient sample collection and processing, reviewed the patient information, interpreted the results and wrote the manuscript. RM – Was involved in the analysis of the data. MT – Performed the IR-spectroscopy measurements. CO – Supervised and organized the genetic analysis of the Italian samples. RZ – Collected, supervised and organized the genetic analysis of the Spanish samples. TS - Was involved in the analysis of the data. BW - Was involved in analysis of the data and reviewing the manuscript. All authors read and approved the final manuscript.
